# Helicobacter pylori infection selectively attenuates endothelial function in male mice *via* exosomes-mediated ROS production

**DOI:** 10.3389/fcimb.2023.1142387

**Published:** 2023-05-18

**Authors:** Linfang Zhang, Xiujuan Xia, Hao Wu, Xuanyou Liu, Qiang Zhu, Meifang Wang, Hong Hao, Yuqi Cui, De-Pei Li, Shi-You Chen, Luis A. Martinez-Lemus, Michael A. Hill, Canxia Xu, Zhenguo Liu

**Affiliations:** ^1^ Center for Precision Medicine and Division of Cardiovascular Medicine, Department of Medicine, University of Missouri School of Medicine, Columbia, MO, United States; ^2^ Department of Gastroenterology, The Third Xiangya Hospital, Central South University, Changsha, China; ^3^ Department of Surgery, University of Missouri School of Medicine, Columbia, MO, United States; ^4^ Dalton Cardiovascular Research Center, University of Missouri, Columbia, MO, United States; ^5^ Department of Medical Pharmacology and Physiology, University of Missouri, Columbia, MO, United States

**Keywords:** Helicobacter pylori, sex difference, endothelial dysfunction, atherosclerosis, reactive oxygen species

## Abstract

**Background:**

Substantial sex differences exist in atherosclerosis. Excessive reactive oxygen species (ROS) formation could lead to endothelial dysfunction which is critical to atherosclerosis development and progression. Helicobacter pylori (H. pylori) infection has been shown to attenuate endothelial function via exosomes-mediated ROS formation. We have demonstrated that H. pylori infection selectively increases atherosclerosis risk in males with unknown mechanism(s). The present study was to test the hypothesis that H. pylori infection impaired endothelial function selectively in male mice through exosome-mediated ROS formation.

**Methods and results:**

Age-matched male and female C57BL/6 mice were infected with CagA+ H. pylori to investigate sex differences in H. pylori infection-induced endothelial dysfunction. H. pylori infection attenuated acetylcholine (ACh)-induced endothelium-dependent aortic relaxation without changing nitroglycerine-induced endothelium-independent relaxation in male but not female mice, associated with increased ROS formation in aorta compared with controls, which could be reversed by N-acetylcysteine treatment. Treatment of cultured mouse brain microvascular endothelial cells with exosomes from H. pylori infected male, not female, mice significantly increased intracellular ROS production and impaired endothelial function with decreased migration, tube formation, and proliferation, which could be prevented with N-acetylcysteine treatment.

**Conclusions:**

H. pylori infection selectively impairs endothelial function in male mice due to exosome-mediated ROS formation.

## 1 Introduction

There are substantial sex differences in many cardiovascular diseases (CVDs) including atherosclerosis, coronary artery disease (CAD), heart failure, cardiac hypertrophy, and stroke (Kander et al., 2017). It is well known that premenopausal women are relatively protected from CVDs when compared to men of similar age, and typically, women are almost 10 years older than men when affected by CAD (Radovanovic et al., 2012). However, the mechanisms for significant sex differences in CVDs have not been well defined.

Gut microorganisms significantly contribute to the development and progression of atherosclerosis and related CVDs (Brown and Hazen, 2018; Catry et al., 2018). Helicobacter pylori (*H. pylori*) is a gram-negative bacterium that colonizes gastric epithelium in a significant portion of the global population (Hooi et al., 2017). Population studies have indicated that *H. pylori* infection is independently associated with the development of CVDs (Kowalski, 2001; Sawayama et al., 2005; Chen et al., 2012; Wang et al., 2012). A meta-analysis with 19,691 study subjects showed that *H. pylori* infection increased the risk of adverse cardiovascular events by 51%, mostly due to myocardial infarction and cerebrovascular disease (Wang et al., 2020). A recent study with 208,196 subjects revealed that *H. pylori* eradication therapy significantly decreased the risk for death and the composite endpoints for CAD in younger patients (< 65 years old) as compared with those without *H. pylori* eradication, while no benefits were observed in older patients (≥65 years old) (Wang et al., 2018). We recently analyzed a database of 17,613 adult patients with carotid ultrasound assessment and a ^13^C-urea breath test for *H. pylori*. We reported that, after adjusting for age, sex, body mass index, lipid profile, hypertension (HTN), diabetes mellitus (DM), and smoking, *H. pylori* infection was an independent risk factor for carotid atherosclerosis in male patients ≤ 50 years, but not in older male or female patients (Zhang et al., 2019). However, how *H. pylori* infection could lead to atherosclerosis only in younger males remains unknown.

Endothelial cell dysfunction resulting from excessive reactive oxygen species (ROS) is an important contributing factor to the pathogenesis of CVDs including HTN and atherosclerosis (Gimbrone and García-Cardeña, 2016). Our recently published data have shown that *H. pylori* infection significantly decreases endothelium-dependent flow-mediated dilatation (FMD) of the brachial artery in young patients without known risk factors as compared to age- and sex-matched healthy volunteers (Xia et al., 2020). The data from animal studies also demonstrated that serum exosomes from patients with *H. pylori* infection significantly reduced endothelial function with decreased cell migration, tube formation, and proliferation *in vitro (* Xia et al., 2020). However, whether there are sex differences in *H. pylori* induced endothelial dysfunction and the underlying mechanisms remain unclear.

Exosomes are bilayer liposomal vesicles from many cells and play important roles in intercellular communications and cell functions through multiple mechanisms including regulation of extra- and intracellular levels of reactive oxygen species (ROS) (Nie et al., 2019; Li et al., 2021; Maldonado et al., 2021; Matsuoka et al., 2021). A recent study indicated that *H. pylori* infection could lead to inflammation and increased systemic ROS production in the vascular wall (Souza et al., 2019; Xia et al., 2022). However, differences between males and females and potential mechanism(s) contributing to sexual dimorphism remain unknown. The present study was therefore designed to test the hypotheses that *H. pylori* infection induces endothelial dysfunction selectively in male but not in female mice through exosome-mediated ROS formation. The objectives of the present study included: 1) to evaluate if there were significant sex differences in endothelial dysfunction with *H pylori* infection; 2) to define the role of ROS in sex differences in endothelial dysfunction with *H. pylori* infection; and 3) to determine the role of exosomes in *H pylori* infection-induced ROS production and endothelial dysfunction.

## 2 Materials and methods

### 2.1 Culture of *H. pylori*

Our previous data suggested that CagA^+^ *H. pylori*, not CagA^-^ *H. pylori*, infection significantly decreased acetylcholine (ACh)-induced aortic relaxation and enhanced early atherosclerosis formation (Xia et al., 2022). Thus, CagA^+^ *H. pylori*, originally isolated from the gastric tissue of a gastric ulcer patient, were used in the present study as described (Xia et al., 2020). Briefly, CagA^+^ *H. pylori* was cultured in Columbia blood agar with 10% sheep blood (Fisher Scientific 50863755, Waltham, MA, USA) with antibiotics (CampyGen sachet, Oxoid) at 37°C under microaerophilic conditions (5% O_2_, 10% CO_2_ and 85% N_2_) for 3-4 days. The CagA^+^ *H. pylori* strain was confirmed using gram staining, biochemical tests, and PCR assay as described (Xia et al., 2020). The bacterial density was determined spectrophotometrically using the optical density (OD) of 600 nm (OD600). An absorbance of 1.0 at OD600 was equivalent to approximately 2x10^8^ colony-forming unit (CFU)/ml (Xia et al., 2020).

### 2.2 Animal models

The animal studies were conducted in compliance with the “Guide for the Care and Use of Laboratory Animals of the US National Institutes of Health”. The study protocols were reviewed and approved by the Institutional Animal Care and Use Committee of the University of Missouri School of Medicine, Columbia, MO, USA (Protocol Number: 10118). Specific-pathogen-free male (4-6-week-old) and age-matched female C57BL/6 wild-type mice (WT) were obtained from Jackson Lab (ME, USA), and were maintained in a constant environment with regulated temperature (20-22°C), humidity (approximately 55%), and a 12/12 h light/dark cycle with ad libitum access to food and water.

After overnight fasting, male and female mice were given oral gavage with 0.2 ml of bacterial suspension (approximate 4×10^9^CFU/ml) or PBS (control) as described (Xia et al., 2020). The presence of *H. pylori* infection was assessed at the end of the experiment using a Rapid Urease Test (RUT) and Giemsa staining of gastric mucosa as described (Xia et al., 2020). Mice were euthanized using >70% CO_2_ exposure under general anesthesia with isoflurane (1.5%) one week and 12 weeks after the last gavage, and the thoracic aortas were collected and prepared for *ex vivo* vascular function studies and ROS measurement as described (Xia et al., 2020).

### 2.3 *Ex vivo* evaluation of vascular function

Thoracic aortae were isolated and carefully prepared under a dissection microscope for ex vivo evaluation of vascular function as described (Xia et al., 2020). Briefly, aortic ring segments (2-3 mm) were mounted horizontally on stainless steel wire hooks of a 4-Channel Myograph System (610M; DMT, Aarhus, Denmark) to measure the isometric aortic contraction and relaxation. The aortic preparations were allowed to equilibrate for 1 hour at an initial tension of 1g in organ bath with 5 ml of Krebs solution (containing 118 mM NaCl, 4.7 mM KCl, 1.2 mM MgSO4, 1.25 mM CaCl2, 1.2 mM KH2PO4, 25 mM NaHCO3, and 11 mM glucose) and continuously bubbled with 95% O_2_ and 5% CO_2_. The aortic rings were first challenged with 50 mM KCl in Krebs solution to determine the maximum contraction. After obtaining a concentration response curve to phenylephrine (PE, 10^-9^-10^-5^M, accumulative) and washing out, the aortic preparations were sub-maximally (70-80% of maximal contraction) using phenylephrine (10^–6^ M) to determine the dose-response curves for endothelium-dependent relaxation to acetylcholine (ACh, 10-9-10-5M, accumulative) and endothelium-independent relaxation to nitroglycerin (NTG, 10-9-10-5M, accumulative).

### 2.4 Exosome preparation and characterization

Mouse serum was collected and to prepare exosomes by centrifuging at 4°C (300 × g for 10 minutes, 2,000xg for 20 minutes, and 10,000xg for 30 minutes) to remove the cells and cell debris as described (Théry et al., 2006; Xia et al., 2020). Exosomes were then obtained from the supernatant by ultracentrifugation (Beckman Coulter, Indianapolis, IN, US) at 100,000×g at 4°C for 70 minutes for two times, and then re-suspended in PBS for identification by morphological characteristics using transmission electron microscopy (TECNAI G2 Spirit; FEI, Hillsboro, OR, US), and size distribution using a Zetasizer Nano ZS (Malvern Instrument, UK).

### 2.5 Cell culture and endothelial function measurement

Mouse brain microvascular endothelial cells (bEND.3) were obtained from ATCC. Cells were maintained in DMEM (Gibco, Grand Island, NY, US) with 10% FBS (Gibco, US), 100 mg/ml streptomycin, and 100 U/ml penicillin at 37°C with 5% CO2 and 95% room air as described (Xia et al., 2022). After 4 hours of exposure to serum exosomes from mice with or without CagA^+^ *H. pylori* infection, bEND.3 cells were tested for ROS production (see details below) and cell function by measuring their migration, tube formation and proliferation as described (Xia et al., 2022).

### 2.6 Administration of N-acetylcysteine

To confirm the role of ROS formation in *H. pylori* infection-induced endothelial dysfunction, the FDA-approved drug N-acetylcysteine (NAC, Sigma-Aldrich, MO, USA), was used both *in vivo* and *in vitro*. For *in vitro* study, NAC (10 mM, final concentration) was added to the culture media of bEnd.3 cells as described (Xia et al., 2020); For *in vivo* study, mice were treated with NAC *via* drinking water (1 mg/ml of NAC) three days before the first gavage with continuation until the end of the experiment as described (Cui et al., 2020).

### 2.7 ROS measurement

ROS levels in the cryostat sections of aorta were evaluated using the indicator Dihydroethidium (DHE, Invitrogen D23107, Waltham, MA, United States) in conjunction with fluorescence microscopy as described (Lau et al., 2013). Briefly, fresh frozen cryostat sections (5 μm) of mouse aortic rings were incubated with 5 μM DHE in dark for 7 min. The cryostat sections were then washed with PBS 3 times at 4°C and the fluorescence images were acquired using an excitation wavelength of 518 nm and an emission wavelength of 606 nm. Fluorescence intensity was analyzed and quantified with ImageJ software.

The levels of intracellular ROS in bEND.3 cells were evaluated using the fluorescent dye 2′7′- dichlorodihydrofluorescein diacetate (H2DCFDA; Invitrogen D399) as described (Guo et al., 2016). Briefly, after 4 hours of treatment with exosomes, cells were incubated with 15uM H2DCFDA staining solution at 37°C in dark for 30 min and washed 3 times with sterile PBS. The fluorescent signal in the cells was examined using a fluorescence microscope, and the fluorescence intensity was evaluated using ImageJ software.

### 2.8 Statistical analysis

The data were presented as means ± standard error (SEM). All data sets were tested for normality using normal quantile plots and Kolmogorov-Smirnov test. One-way ANOVA (analysis of variance) followed by *post-hoc* conservative Tukey’s tests was used for three or more groups of data with normal distributions. A two-tailed unpaired t-test was used for the analysis of two groups of data with normal distribution and equal variance. SPSS statistical software (22.0 for Windows; IL, USA) was used for the analysis, and a *p*<0.05 was considered statistically significant.

## 3 Results

### 3.1 *H. pylori* infection selectively impaired endothelial function in male mice

There could be multiple reasons for sex differences in endothelial dysfunction associated with *H. pylori* infection including the ability of gastric colonization. Using age-matched male and female C57BL/6 mice, we observed that there was no difference in *H. pylori* infection rate between male and female mice. After three daily doses of *H. pylori* inoculums, all male and female mice (100%) were successfully infected with *H. pylori*, as verified with both RUT and Giemsa staining of gastric mucosa (**Supplemental Figure 1**). Thus, there was no significant difference in the efficiency of *H. pylori* infection between the males and females.

**Figure 1:**
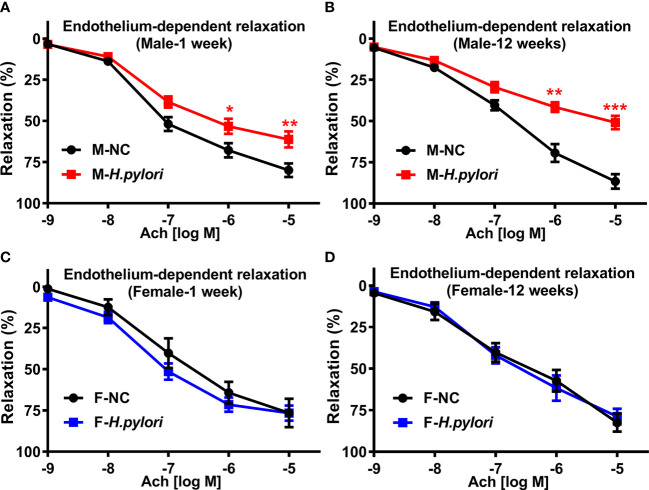
*H. pylori* infection selectively resulted in significant endothelial dysfunction in male C57BL/6 mice. Endothelium-dependent aortic relaxation to ACh was significantly attenuated in male C57BL/6 mice **(A, B)**, not in females **(C, D)** with acute (1 week) or chronic (12 weeks) *H. pylori* infection over the control. NC, normal control; Ach, acetylcholine; Data are shown as mean ± SEM. *P<0.05, **P<0.01, ***P<0.001 using t-test, n=8-10 mice for each group at each time point.

To determine if there was a sex difference in the effect of *H. pylori* infection on endothelial function, mouse models of both acute (1 week) and chronic (12 weeks) *H. pylori* infection were created using age-matched male and female C57BL/6 mice infected with PBS as control. RUT and Giemsa staining confirmed that all mice were successfully infected with *H pylori*. As shown in **Figure 1**, endothelium-dependent aortic relaxation was significantly impaired in male mice with both acute and chronic *H. pylori* infection as compared with their controls (**Figure 1**). There was no significant difference in endothelium independent aortic relaxation in response to NTG in male mice with either acute or chronic *H. pylori* infection (**Supplemental Figure 2**). However, no significant decrease in ACh-induced relaxation (**Figure 1**) or NTG-induced relaxation (**Supplemental Figure 2**) was observed in female mice with acute or chronic *H. pylori* infection. No significant difference in aortic contraction to PE was observed between male and female mice with or without *H. pylori* infection (**Supplemental Figure 3**).

### 3.2 ROS production was selectively increased in the aorta of male mice with *H. pylori* infection

**Figure 2:**
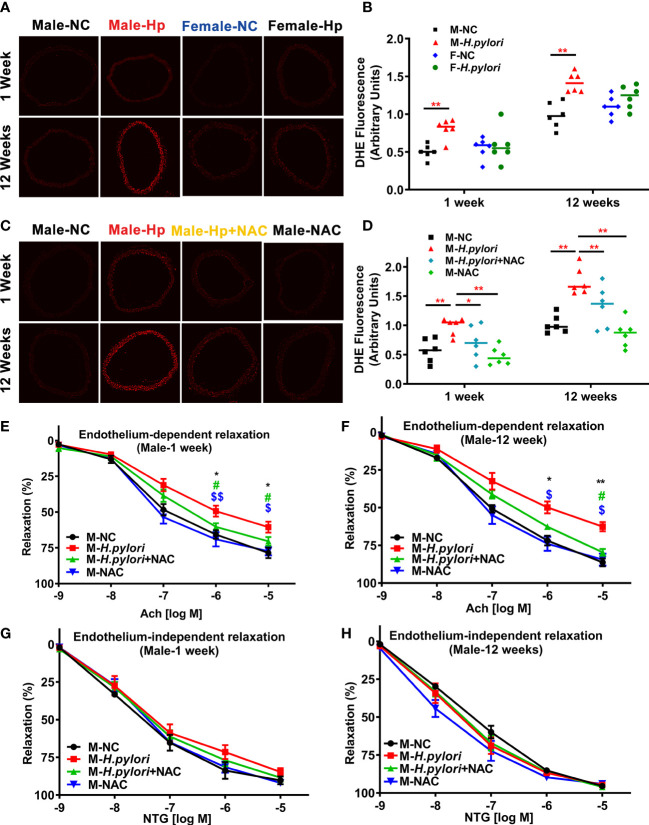
*H. pylori* infection selectively impairs endothelial function in male mice *via* excessive ROS formation. Representative fluorescent images **(A)** and quantitative analysis of aortic ROS **(B)** in male and female C57BL/6 mice with *H. pylori* infection or PBS control (**P<0.01 by t-test). Treatment with NAC effectively prevented aortic ROS production **(C, D)** (*P<0.05, **P<0.01 by one-way ANOVA with Bonferroni’s test or Kruskal-Wallis test with Dunn’s *post hoc* test) and preserved aortic relaxation to Ach **(E, F)** in male C57BL/6 mice with acute (1 week) or chronic (12 weeks) *H. pylori* infection, without change in endothelium-independent aortic relaxation to NTG **(G, H)**. *P<0.05, **P<0.01 (vs NC), ^#^ *P*<0.05 (vs *H. pylori* + NAC); ^$^ *P*<0.05, ^$$^ *P*<0.01 (vs NAC) by one-way ANOVA with Bonferroni’s test or Kruskal-Wallis test with Dunn’s *post hoc* test. NC, normal control; Hp, *H. pylori*; Ach, acetylcholine; NAC, N-acetylcysteine; NTG, nitroglycerin. Data are shown as mean ± SEM; n=8-10 mice for each group at each time point.

To determine the role of ROS in *H. pylori* infection-induced endothelial dysfunction, aortic ROS levels were evaluated in age-matched male and female C57BL/6 mice with and without *H. pylori* infection. It was observed that *H. pylori* infection significantly attenuated aortic relaxation in response to ACh in male, but not female, C57BL/6 mice, associated with a significant elevation of ROS levels in the aorta in male mice at both 1 week and 12 weeks post infection over the controls (**Figure 2**). To further evaluate the role of ROS in *H. pylori* infection-induced endothelial dysfunction, male C57BL/6 mice were treated with NAC *via* drinking water. As expected, treatment of mice with NAC indeed effectively prevented excessive aortic ROS production (**Figure 2**) with preserved aortic relaxation to ACh in male mice (**Figure 2**) without significant changes in aortic relaxation to NTG (**Figure 2**) after 1 week and 12 weeks of *H. pylori* infection.

### 3.3 Serum exosome from male mice with *H. pylori* infection attenuated endothelial function via excessive ROS formation in vitro

**Figure 3:**
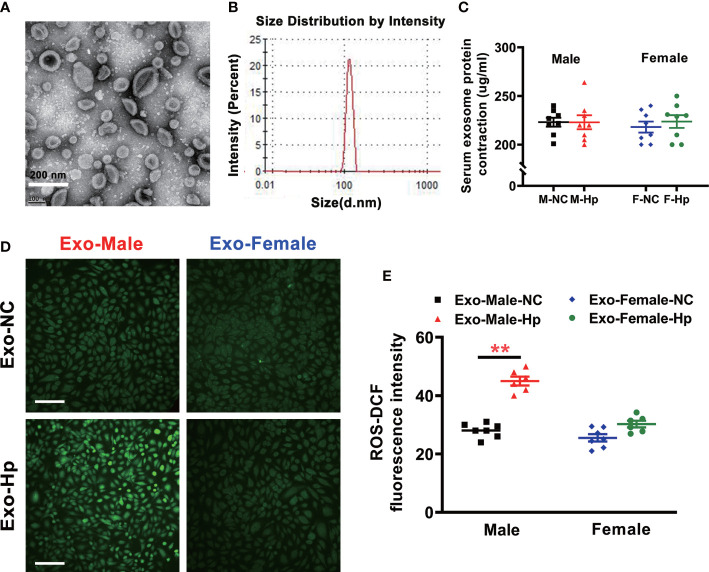
Exosomes from the serum of male mice with *H. pylori* infection increased intracellular ROS level *in vitro*. Exosomes isolated from mice with *H. pylori* infection exhibited typical exosome characteristics with unique morphologies as shown on transmission electron microscopy **(A)** and size distribution **(B, C)**There was no significant difference in serum exosomes protein levels between male and female mice infected with *H. pylori*; **(D, E)** Co-culture of bEND.3 cells with exosomes from the serum of male mice, not from the serum of female mice, with *H. pylori* infection significantly enhanced the levels of intracellular ROS in bEND.3 cells. NC, negative control; Hp, *H.* pylori; Exo, Exosomes; Exo-NC, Exosomes from C57BL/6 mice with PBS gavage; Exo-H. pylori, Exosomes from C57BL/6 mice with *H.* pylori *infection.* **P < 0.01 by t-test. Data are shown as mean ± SEM; n=7-8 independent experiments for every measurement. Scale bars = 100 μm.

**Figure 4:**
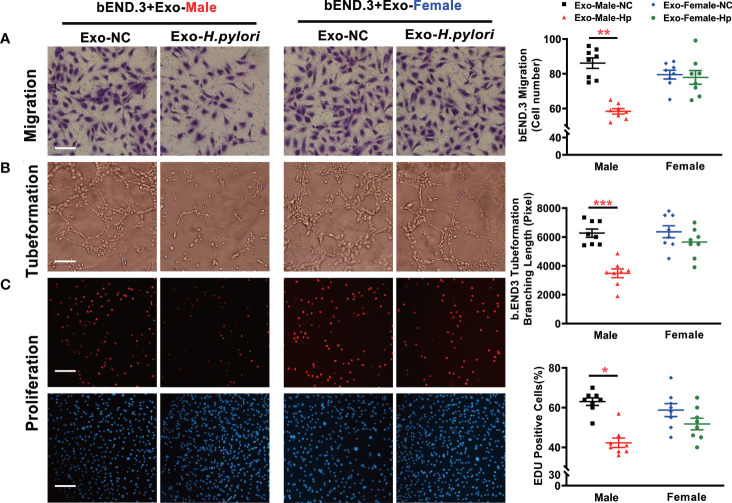
Exosomes from the serum of male mice with *H. pylori* infection attenuated endothelial function *in vitro*. Treatment of bEND.3 with serum exosomes from *H. pylori* infected male C57BL/6 mice, but not female mice significantly inhibited the endothelial function with decreased migration **(A)**, tube formation **(B)**, and proliferation **(C)**. NC, negative control; Hp, *H.*pylori; Exo, Exosomes; Exo-NC, Exosomes from C57BL/6 mice with PBS gavage; Exo-H. pylori, Exosomes from C57BL/6 mice with *H.* pylori *infection.* *P< 0.05, **P < 0.01, ***P < 0.001 by t-test. Data are shown as mean ± SEM; n=6-7 independent experiments for every measurement. Scale bars **(A)** = 25 μm; Scale bars **(B)** = 10; Scale bars **(C)** = 100 μm.

**Figure 5:**
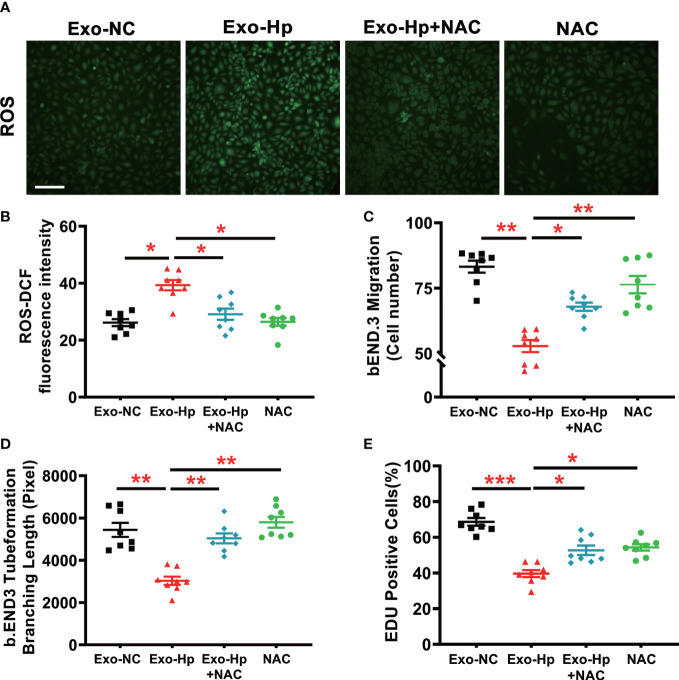
**NAC** treatment effectively prevented ROS formation and preserved endothelial function *in vitro*. Treatment of bEND.3 cells with NAC effectively attenuated the levels of intracellular ROS **(A, B)** and maintained the function of bEND.3 cells **(C–E)** co-cultured with serum exosomes from *H. pylori* infected male mice. Exo-NC, exosomes from control male mouse serum; Exo-Hp, Exosomes from *H. pylori* infected male mouse serum; NAC, N-acetylcysteine. Data are shown as mean ± SEM; *P<0.05; **P<0.01; ***P<0.001 by one-way ANOVA. n=8 independent experiments for every measurement. Scale bar **(A)** = 100 μm.

To determine how *H. pylori* infection could impair endothelial function, we tested the hypothesis that *H. pylori* infection selectively impaired endothelial function in males through exosomes-mediated mechanisms. Serum exosomes from both male and female mice were isolated and identified by morphology (**Figure 3**) and molecular size distribution (**Figure 3**). There was no significant difference in serum exosomes protein levels between male and female mice infected with *H. pylori* (**Figure 3**). However, treatment of mouse brain microvascular endothelial cells (bEND.3) with exosomes from the serum of male mice with *H. pylori* infection, but not from the serum of female mice with *H. pylori* infection, significantly increased intracellular ROS formation (**Figure 3**) in bEND.3 cells and inhibited their function with decreased migration (**Figure 4**), tube formation (4B), and proliferation (4C) compared with control. NAC treatment (added to the culture media) effectively attenuated excessive intracellular ROS level (**Figure 5**) and maintained the function of bEND.3 cells (**Figures 5D**) in the presence of exosomes from male mice with *H. pylori* infection.

## 4 Discussion

In the present study, we demonstrated: 1) *H. pylori* infection selectively impaired endothelial function in male mice, but not female mice; 2) *H. pylori* infection selectively increased aortic ROS formation in male mice, and blocking excessive ROS formation with NAC treatment effectively prevented endothelial dysfunction in male mice with *H. pylori* infection; 3) Exosomes from the serum of male mice with *H. pylori* infection, but not from the serum of female mice, significantly enhanced the level of intracellular ROS and induced endothelial function, which could be reversed by NAC treatment. These data indicate that *H. pylori* infection selectively impairs endothelial function in male mice *via* exosome-mediated excessive ROS formation.

Major differences between men and women exist in epidemiology, pathophysiology, and symptomatology of CVDs, such as coronary artery disease, hypertension, and atherosclerosis. However, the mechanisms for sex differences in CVDs remain largely unknown. It is widely believed that estrogen is responsible for the protection of women from CVDs in the premenopausal age (Murphy and Steenbergen, 2007; Miller and Duckles, 2008). However, hormone replacement therapy has failed to decrease major adverse cardiovascular events in clinical studies (Duvernoy et al., 2007) which suggests that there are still other residual risk factors that have not been yet defined and may contribute significantly to the observed sex differences in vascular endothelial dysfunction.

Studies have demonstrated that *H. pylori* infection, especially with a virulence factor CagA-positive strain (CagA^+^), is independently associated with intima-media thickness and atherosclerosis risk (Mayr et al., 2003; Yang et al., 2019), especially in males. An epidemiological study reported that *H. pylori* infection could trigger more metabolic abnormalities and atherosclerosis in men than in women (Longo-Mbenza et al., 2007). Consistent with this, a Japanese study with 6,289 subjects reported a higher LDL-C level and a lower HDL-C level with *H. pylori* infection in men, not in women (Satoh et al., 2010). In the present study, we observed that *H. pylori* infection selectively impaired endothelial function in male mice, not females. Similarly, the data from our previous study with a database of 17,613 adult patients has revealed that *H. pylori* infection is an independent risk factor for carotid atherosclerosis and increase of carotid intima-media thickness in male patients of 50 years old or younger, but not in female subjects (Zhang et al., 2019). The finding from the present study may explain the clinical observation that *H pylori* infection selectively increases the risk for atherosclerosis in male patients, not in females, in view of the important role of endothelial function in the development and progression of atherosclerosis. However, the underlying mechanisms still require further investigations at cellular and molecular levels.

Endothelial dysfunction is an early hallmark of vascular diseases and atherosclerosis and may provide insights into sex differences in CVDs. Inflammation and oxidative stress are critical to the development and progression of endothelial dysfunction (Marchio et al., 2019). Accumulating evidence demonstrates that *H. pylori* infection potentially triggers ROS formation and oxidative stress (Ernst, 1999), leading to endothelial dysfunction. *H. pylori* infection could increase ROS levels in endothelial cells through multiple mechanisms. It has been reported that *H. pylori* infection increases the levels of serum free fatty acids and leptin, enhancing ROS formation by increasing beta oxidation and oxidation of the resultant acetyl-CoA through TCA cycle (Azuma et al., 2001; Yamagishi et al., 2001). Moreover, *H. pylori* infection could increase serum myeloperoxidase, an oxidative enzyme in phagocytes that is secreted at inflammatory sites in vascular wall to increase oxidative stress (Nazligul et al., 2011). It is also reported that exosomes from *H. pylori*-infected gastric mucosa could carry soluble bacterial antigens (CagA, VacA, urease, and neutrophil activating factor A), viral proteins and other pathogenic factors into circulation, leading to vascular inflammation and oxidative stress (Shimoda et al., 2016; Li et al., 2019; Yang et al., 2019). Mechanistically, the endothelium is directly involved in a number of pathophysiological processes through its dynamic interactions with blood components and extracellular vesicles. Exosomes are small subcellular organelles of 30 to 200 nm (average 40-100 nm) in diameter that have the same topology as cells and contain a variety of proteins, lipids, nucleic acids, microRNAs, and glycoconjugates (Valadi et al., 2007; Mittelbrunn et al., 2011; Borges et al., 2013; Pegtel and Gould, 2019). These small vesicles are known to be critically involved in the development of endothelial dysfunction and CVDs by extensive cell-cell communications through transport of a wide spectrum of bioactive constituents inside the vesicles (Valadi et al., 2007; Mittelbrunn et al., 2011). Our previous study, using PKH67-labeling technique and 3-D confocal microscopy, showed that CagA-containing exosomes could be transported into HUVECs shortly after co-culture *in vitro*, leading to a significant reduction of the function of HUVECs with decreased migration, proliferation, and tube formation through increased ROS production (Xia et al., 2022).

However, the sex differences and the potential mechanisms in *H pylori* infection-induced ROS formation and related endothelial dysfunction are rarely reported. In the present study, we observed that *H pylori* infection significantly increases aortic ROS production selectively in male mice, not in female mice, through exosomes-mediated mechanism(s). Exosomes from the serum of male mice, not from the serum of female mice, with *H. pylori* infection could significantly increase intracellular ROS levels and inhibit endothelial function. This finding may provide an explanation and potential mechanism for the sex difference in CVDs for patients with *H pylori* infection. However, further research is still needed to reveal the molecular mechanisms for sex differences in exosomes in *H. pylori* infection-associated endothelial dysfunction.

Studies have shown that there are significant differences in exosomes and their cargo contents between males and females in various disease conditions (Kolhe et al., 2017; Dekker et al., 2020; Ibáñez et al., 2020). A recent study (Bæk et al., 2016) has shown that smoking may lead to differential changes in the protein profiles in extracellular vesicles (EVs) in females versus males. Although there were no significant differences between healthy males and females, plasma EVs from male smokers contained higher plasma levels of CD171 (L2CAM), PD-L1 (programming cell death ligand 1), and TSG101 (tumor susceptibility gene 101) than female smokers and male non-smokers. On the other hand, the EV levels of AREG (amphiregulin), MUC1 (CA15-3), CD146 (MUC18), the alanine aminopeptidase CD13 and TSG101 were significantly decreased in female smokers compared with female non-smokers, while no changes in these proteins were observed in male smokers. In addition, males had lower EV amounts of AREG and MUC1 with advanced age. However, whether there are significant differences in the protein profiles of plasma exosomes between males and females with *H. pylori* infection has not been reported yet. In the present study, we observed that co-culture of bEND.3 cells with exosomes from the serum of male mice with H. pylori infection, not from the serum of females resulted in a significant increase in the levels of intracellular ROS, although there was no significant difference in serum exosomes protein levels between male and female mice infected with *H. pylori*. The mechanism(s) for increased ROS level in endothelial cells treated with serum exosomes selectively from male, not female, mice with *H. pylori* infection is unclear at this point. However, the cargo contents of serum exosomes between male and female mice infected with *H. pylori* may be significantly different, thus contributing to the sex differences in intracellular ROS levels in endothelial cells. Further mechanistic studies are needed to determine how serum exosomes from male mice with *H. pylori* infection increases ROS production and endothelial dysfunction.

The mechanisms for *H. pylori* infection-induced ROS production and endothelial dysfunction are complex and likely multi-factorial. In addition to CagA-containing exosomes-mediated mechanism, increased productions of inflammatory cytokines could lead to increased intracellular ROS production and endothelial dysfunction, as well as sex differences in ROS levels in endothelial cells. Indeed, *H. pylori* infection is reported to increase the productions of inflammatory cytokines IL-6 and IL-8 (especially IL-8) in endothelial cells (Innocenti et al., 2002; Tafreshi et al., 2018). It is also reported that exosomes derived from *H. pylori*-infected macrophages enhance the expressions of inflammatory cytokines including TNF-α, IL-6, and IL-23 (Wang et al., 2016). Further studies are needed to determine if there is a significant sex difference in the productions of inflammatory cytokines in response to *H. pylori* infection and their potential contributions to sex differences in intracellular ROS production and endothelial dysfunction.

It is of significant clinical concern that CVDs remain the leading the cause of mortality and morbidity globally, the numbers of deaths from CVDs have been steadily increasing every year since 2010 especially for males without a clearly defined mechanism(s) (Pearson-Stuttard et al., 2016; Andersson and Vasan, 2018; Tsao et al., 2022). Clinical and epidemiological studies suggest that *H. pylori* infection could contribute to significant endothelial dysfunction and the development of premature atherosclerosis in young male patients without a clear explanation. In the present study, we demonstrated that NAC treatment effectively attenuated *H. pylori* infection-induced ROS production and preserved endothelial function in male mice. However, it is important to confirm the findings in human subjects with large randomized clinical studies.

## 5 Conclusions

We have demonstrated that *H. pylori* selectively impairs endothelial function in male but not female mice through a mechanism that involves exosome-mediated excessive ROS formation.

## Data availability statement

The raw data supporting the conclusions of this article will be made available by the authors, without undue reservation.

## Ethics statement

The animal study was reviewed and approved by Institutional Animal Care and Use Committee of the University of Missouri School of Medicine, Columbia, MO, USA.

## Author contributions

ZL conceived the idea and designed the study. LZ, XX, HW, XL, HH, YC, D-PL, S-YC, and QZ performed the experiments, collected the data, and conducted the data analysis. LZ, XX and MW contributed to the isolation and characterization of exosomes. LZ, XX, LM-L, and MH were involved in vascular function studies. LZ drafted the manuscript. D-PL, S-YC, LM-L, CX, MH, and ZL critically reviewed and interpreted the data and modified the manuscript. All authors contributed to the article and approved the submitted version.
