# Supplementary material for: Helicobacter pylori infection selectively attenuates endothelial function in male mice via exosomes-mediated ROS production
Source: Front Cell Infect Microbiol. 2023 May 18;13:1142387. doi: 10.3389/fcimb.2023.1142387 (PMC10233065; doi:10.3389/fcimb.2023.1142387)
Supplement: Supplementary file 1 [file DataSheet_1.docx]

Supplementary Material

Helicobacter pylori infection selectively impairs endothelial function in male mice through exosome-mediated ROS formation

Linfang Zhang^1,2†^, Xiujuan Xia^1,2†^, Hao Wu^1^, Xuanyou Liu^1^, Qiang Zhu^1^, Meifang Wang^1^, Hong Hao^1^, Yuqi Cui^1^, De-Pei Li^1^, Shi-You Chen^3^, Luis A. Martinez-Lemus^1,4,5^, Michael A. Hill^4,5^, Canxia Xu^2*^, Zhenguo Liu^1*^

^1^Center for Precision Medicine and Division of Cardiovascular Medicine, Department of Medicine, University of Missouri School of Medicine, Columbia, MO, United States

^2^Department of Gastroenterology, the Third Xiangya Hospital, Central South University, Changsha, China

^3^Department of Surgery, University of Missouri School of Medicine, Columbia, MO, United States

^4^Dalton Cardiovascular Research Center, University of Missouri, Columbia, MO, United States

^5^Department of Medical Pharmacology and Physiology, University of Missouri, Columbia, MO, United States

**^†^**These two contributed to the work equally.

*** Correspondence:**Zhenguo Liu, MD, PhD, Division of Cardiovascular Medicine, Center for Precision Medicine, University of Missouri School of Medicine, 1 Hospital Drive, CE306, Columbia, Missouri 65212, USA. Tel: 573-884-3278.Email: [liuzheng@health.missouri.edu](mailto:liuzheng@health.missouri.edu). Or Canxia Xu, MD. Department of Gastroenterology, Third Xiangya Hospital of Central South University, 138 Tongzipo Road, Changsha 410013, Hunan, China, Email: xucanxia2000@163.com.

# Supplementary Figures


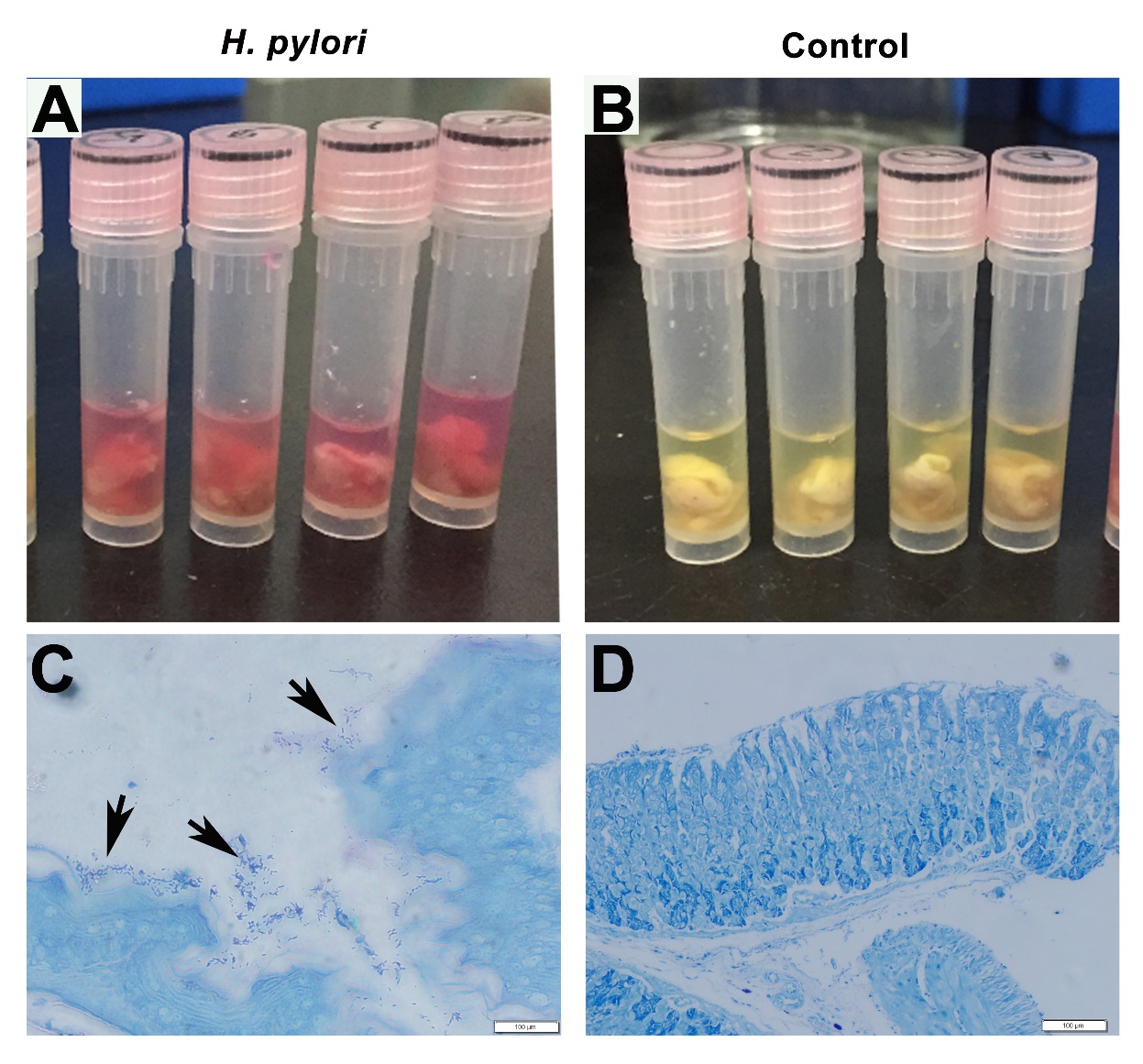
**Supplemental Figure 1. The colonization of *H. pylori* in mouse mucosa tissues.** Mice were tested for *H. pylori* infection using Rapid Urease Test (RUT) and pathological Giemsa staining 7 days after the last intragastric gavage. RUT were reactive positively and turn from yellow to red in mouse stomach with *H. pylori* infection(**A**), but not in PBS control group(**B**). (**C,D**) Geimsa staining showed the characteristic spiral-shaped *H. pylori* organisms (black arrows) were present only in mouse stomach with *H. pylori* infection.


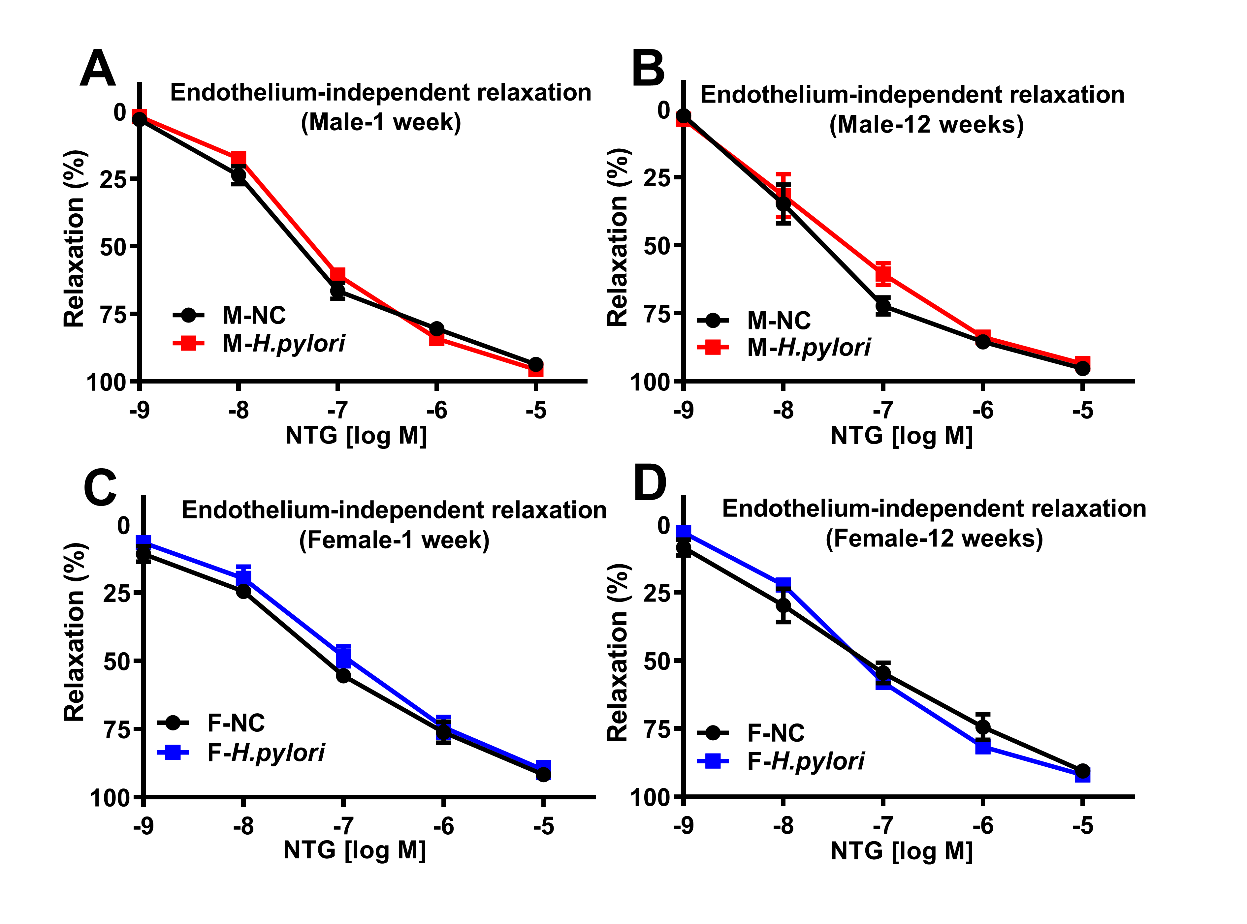


**Supplemental Figure 2. Infection with *H. pylori* had no effect on endothelium-independent relaxation.** Aortas from both male (**A,B**) and female (**C,D**) mice with one week or 12 weeks of *H. pylori* infection exhibited no change in nitroglycerin (NTG)-induced endothelium-independent relaxation responses compared with controls. Data are presented as mean ± SEM. NC: normal control; NTG: nitroglycerin. N=8-10 mice for each group at each time point.

**
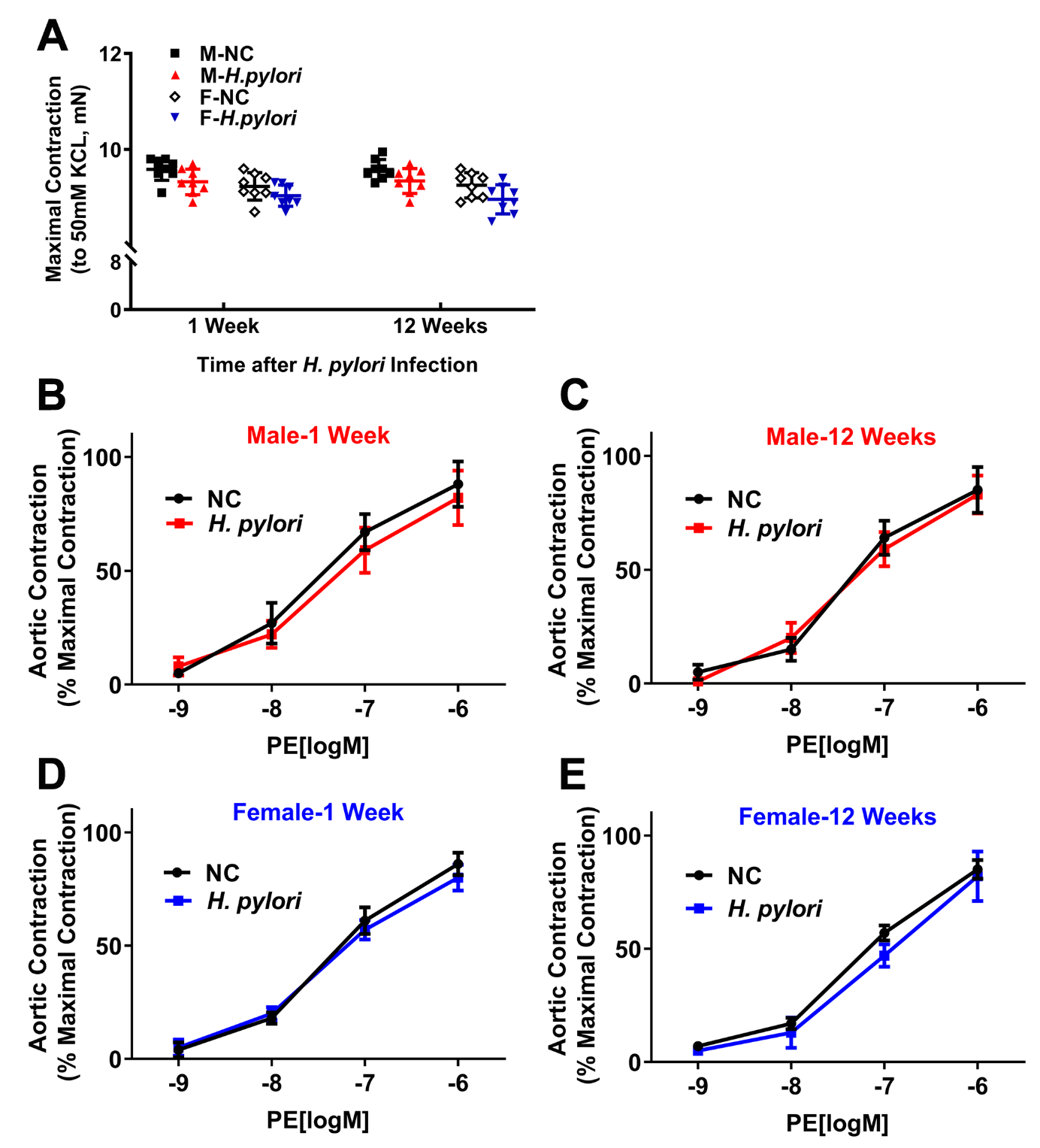
Supplemental Figure 3. Infection with CagA^+^ *H. pylori* had no effect on aortic contraction to either potassium chloride or phenylephrine (PE).** (**A**) Infection with CagA^+^ *H. pylori* had no effect on the maximal aortic contractile response to KCl (50 mM). Aortic preparations from both male (**B, C**) and female (**D, E**) mice with one week or 12 weeks of *H. pylori* infection exhibited no change in their contraction in response to the cumulative dosing of PE as compared with the controls. NC: normal control; PE: phenylephrine. Values are expressed as mean ± SEM. N=8-10 mice for each group at each time point.
